# Case Report: Insulinoma Co-Existing With Type 2 Diabetes – Advantages and Challenges of Treatment With Endoscopic Ultrasound-Guided Radiofrequency Ablation

**DOI:** 10.3389/fendo.2022.957369

**Published:** 2022-07-22

**Authors:** Johnny Yau-Cheung Chang, Chariene Shao-Lin Woo, David Tak-Wai Lui, Matrix Man-Him Fung, Alan Chun-Hong Lee, Eunice Ka-Hong Leung, Yu-Cho Woo, Wing-Sun Chow, Karen Siu-Ling Lam, Kathryn Choon-Beng Tan, Chi-Ho Lee

**Affiliations:** ^1^ Department of Medicine, University of Hong Kong, Hong Kong, Hong Kong SAR, China; ^2^ Department of Surgery, University of Hong Kong, Hong Kong, Hong Kong SAR, China

**Keywords:** insulinoma, type 2 diabetes, continuous glucose monitoring, endoscopic ultrasound, radio frequency ablation

## Abstract

The coexistence of insulinoma and type 2 diabetes is rare and the diagnostic process is often challenging. Continuous glucose monitoring system devices, which are more readily available nowadays, provide a useful tool for the diagnosis and evaluation of treatment response. Curative surgery is often the mainstay of treatment for insulinoma. Here, we report a Chinese patient with insulinoma diagnosed simultaneously with type 2 diabetes. His insulinoma was managed with endoscopic ultrasound guided-radiofrequency ablation (EUS-RFA) and the patient achieved complete resolution of hypoglycaemic episodes. The case illustrates that while EUS-RFA is an emerging non-invasive treatment modality for pancreatic lesions, limitations exist especially when histological confirmation is essential.

## Introduction

Insulinoma is an uncommon neuroendocrine tumour (NET) with an incidence of 4 cases per 1 million person-years (1). Although the clinical presentation of insulinoma and type 2 diabetes (T2D) may seem contradictory, with the former predominates with recurrent refractory hypoglycaemia while the latter is characterized by fasting and post-prandial hyperglycaemia, their coexistence has been infrequently reported (2–5). In a retrospective study conducted by the Mayo clinic, out of the 313 cases of insulinoma over a 65-year period, there was only one case of insulinoma diagnosed in the background of pre-existing T2D (3). Moreover, in the majority of these cases, insulinoma developed in patients whose T2D has been diagnosed for some time. Here, we describe a Chinese patient with an uncommon presentation of insulinoma diagnosed simultaneously with T2D. The insulinoma was successfully treated with endoscopic ultrasound (EUS)-guided radiofrequency ablation (RFA), an emerging non-operative alternative treatment of pancreatic tumours (6). This report discussed the benefits and limitations of EUS-RFA as a definitive treatment of insulinoma.

## Case Report

A 72-year-old man, who had history of hypertension, hyperlipidaemia, coronary artery disease with percutaneous coronary intervention performed in 2018, presented with recurrent hunger sensation in the morning. In retrospect, in the past 3 years prior to his presentation, he had repeated measurements of low fasting glucose down to 3 mmol/L, together with elevated glycated haemoglobin (HbA1c) levels above the diabetic range in pre-clinic blood tests (**Table 1**). His serum fructosamine was 301 umol/L (Ref: 219 – 319 umol/L). His haemoglobin and estimated glomerular filtration rate (eGFR) were 14.2 g/dL and 56ml/min/1.73m^2^, respectively. His morning cortisol was 483nmol/L (Ref: >130 nmol/L). He had never been put on any anti-diabetic agent, and he denied use of herbs, steroids or over-the-counter medications. When he was admitted for further endocrine assessments, the patient was noted to have recurrent fasting hypoglycaemia with capillary blood glucose readings around 2 – 4 mmol/L, but reaching up to 14 mmol/L in the late afternoon. Biochemistry tests confirmed endogenous hyperinsulinaemic hypoglycaemia: fasting plasma glucose was 2.5 mmol/L with a paired serum insulin level of 26 mIU/L, C-peptide 1.2 nmol/L, beta-hydroxybutyric acid <0.05mmol/L and growth hormone 2.7 ng/ml (Ref: <8ng/ml). Urine toxicology for sulphonylurea was negative. Although anti-insulin antibody was not checked, serial dilution of the specimen did not suggest the presence of these autoantibodies.

**Table 1:** Serial HbA1c and fasting glucose levels in the past 3 years prior to the clinical presentation.

	12/2017	7/2018	1/2019	4/2019	8/2019	2/2020
HbA1c (%)	6.8	6.7	6.9	6.7	6.9	7.4
FG (mmol/L)	3.2	5.8	3.1	4.3	3.6	2.2

HbA1c, glycated haemoglobin; FG, fasting glucose.

**Figure 1:**
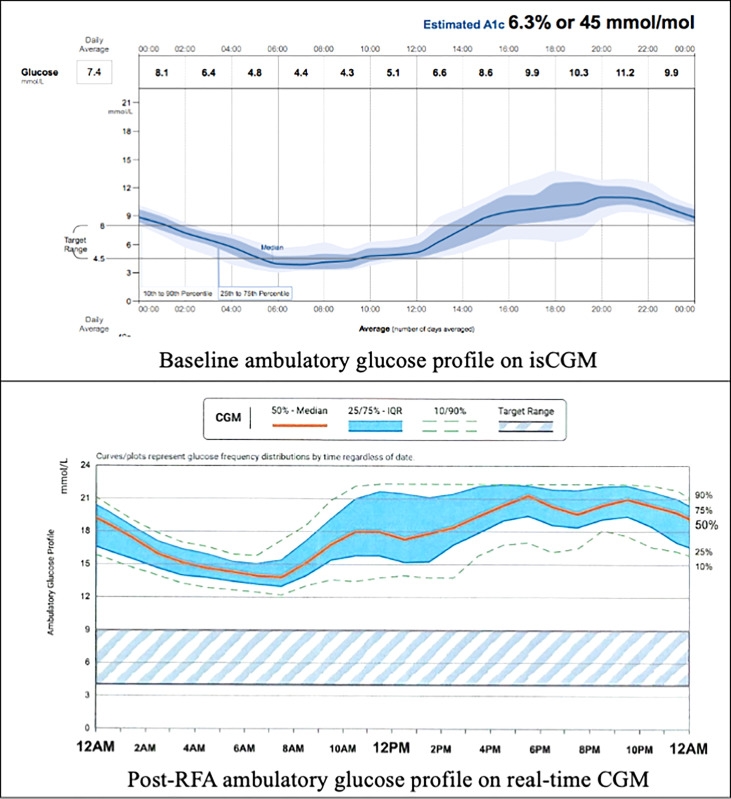
Baseline and post-RFA ambulatory glucose profile. isCGM, intermittently scanned continuous glucose monitoring; RFA, radiofrequency ablation; CGM, continuous glucose monitoring.

In order to comprehensively evaluate his glycaemic profile, an intermittently scanned continuous glucose monitoring (isCGM) device (FreeStyle Libre, 14 Day System) was applied. The ambulatory glucose profile revealed recurrent hypoglycaemic episodes during late evening and early morning which correlated with his hypoglycaemic symptoms. However, he also had significant postprandial glucose excursions with readings reaching above 13.0mmol/L after meals. (**Figure 1**) Overall, only 42% of time was within the pre-set glucose range between 4.5 mmol/L and 8.0 mmol/L, while 41% and 17% of the time his glucose readings were above and below target, respectively.

**Figure 2:**
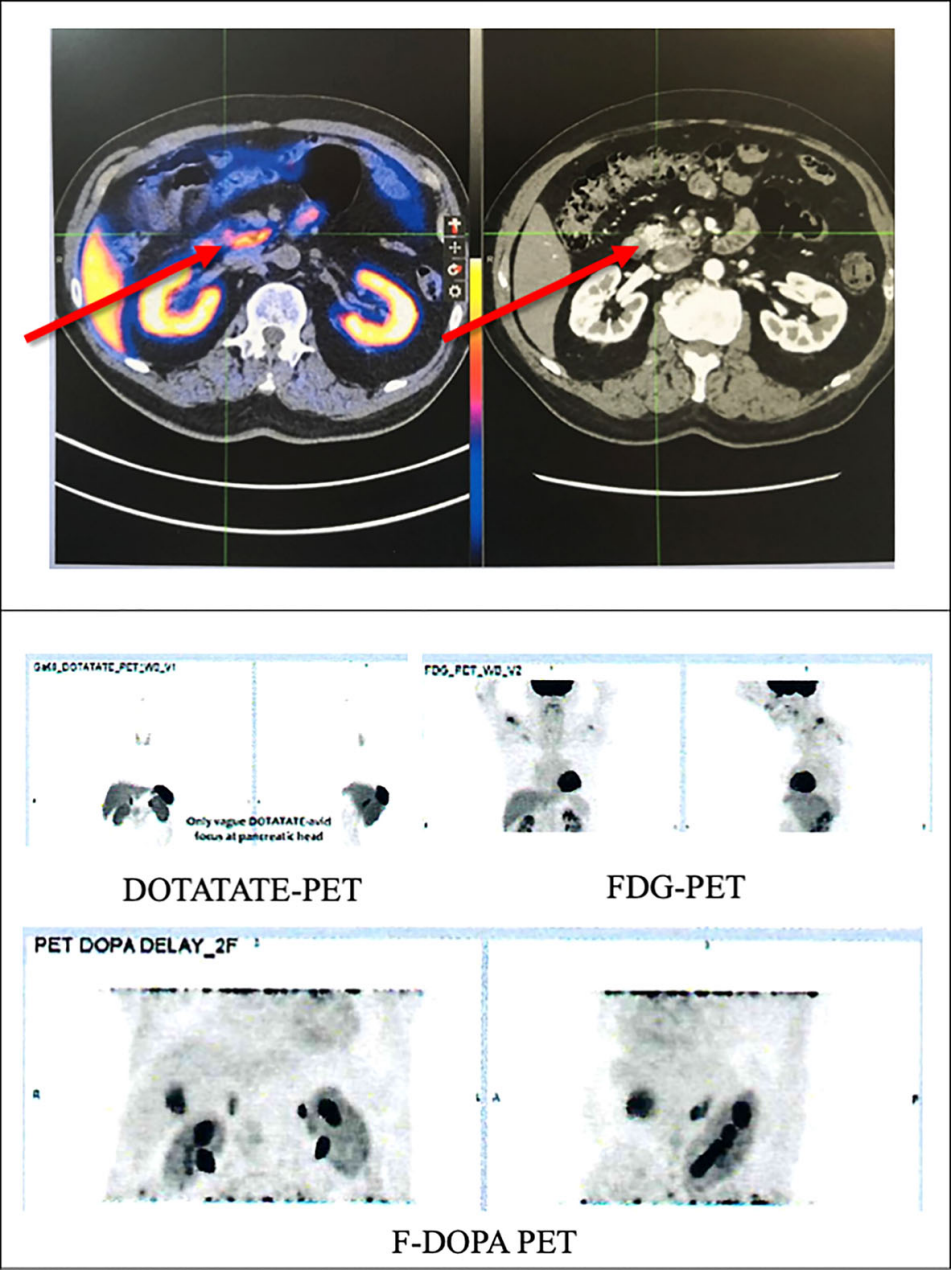
PETCT scan of the patient showing a tumour in the pancreatic head with vague DOTATATE avidity (red arrows) but without FDG or F-DOPA avidity. FDG, ^18^F-fluorodeoxyglucose; F-DOPA, ^18^F-fluorolevodopa (F-DOPA); PETCT, positron emission tomography-computed tomography.

A contrast computed tomography (CT) of his pancreas showed a 1.2x0.9cm arterial enhancing lesion at the pancreatic head, which was isodense on portal venous and delayed phases. The remaining pancreas appeared mildly atrophic but was otherwise unremarkable. Subsequently, an ^18^F-fluorodeoxyglucose (FDG), ^68^Ga-DOTATATE and ^18^F-fluorolevodopa (F-DOPA) positron emission tomography-computed tomography (PETCT) revealed a 1.26x0.94cm (SUV max 9.7) NET at the pancreatic head with vague DOTATATE avidity but without FDG or F-DOPA avidity. No other lesions were detected. (**Figure 2**)

**Figure 3:**
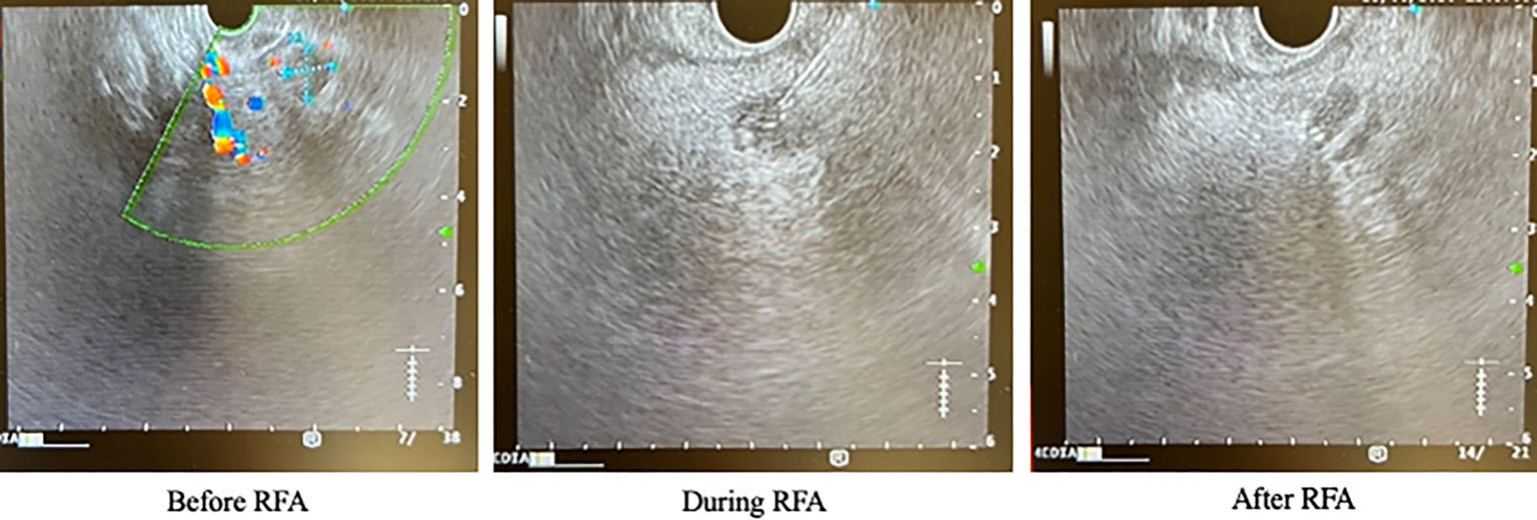
Endoscopic ultrasound images of the patient before, during and after RFA. RFA, radiofrequency ablation.

**Figure 4:**
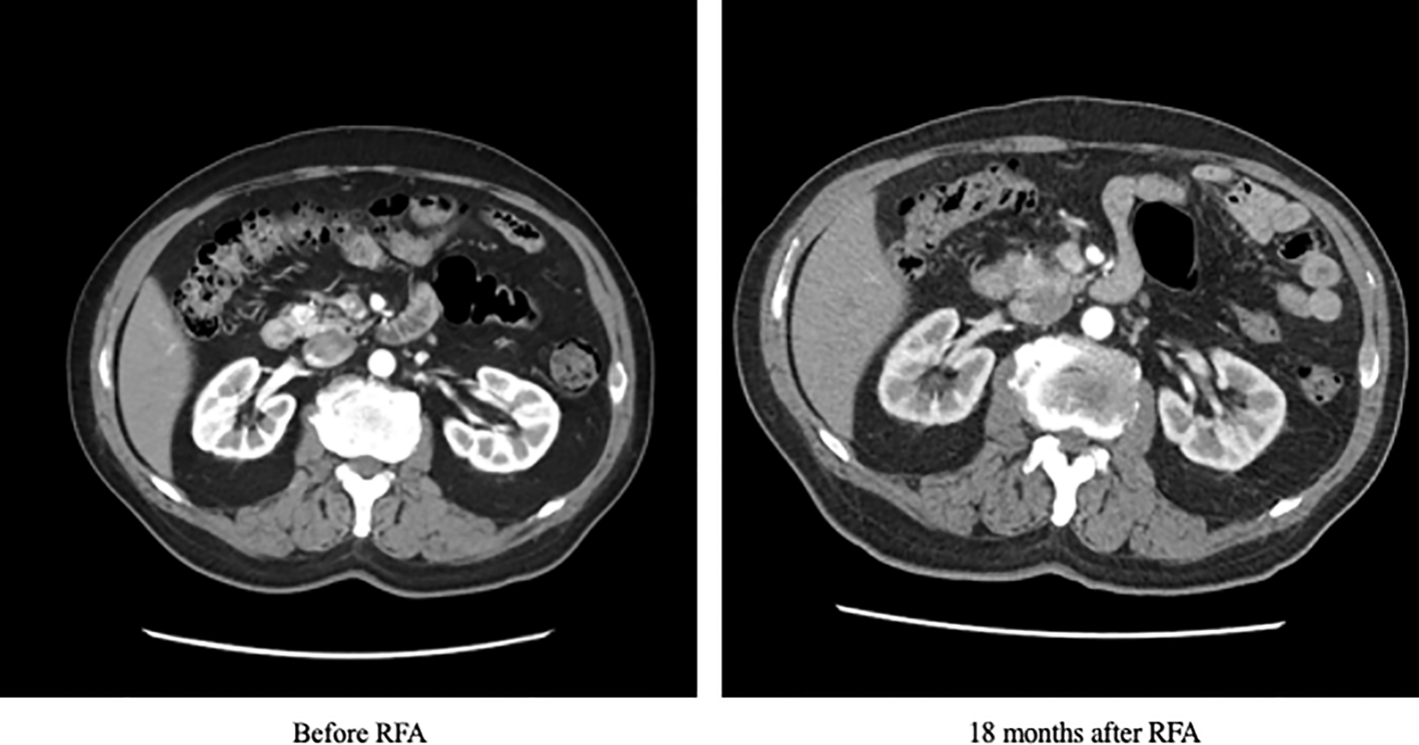
Contrast CT scan of the patient at baseline and 18 months after RFA. CT, computed tomography; RFA, radiofrequency ablation.

The patient was initially managed with dietary modification including the use of corn-starch overnight. He then underwent EUS-guided RFA to the pancreatic head lesion. During the procedure, a 19-gauge EUSRA (Taewoong, USA) needle was inserted to the lesion. The tumour was ablated with 50 watts/cycle for 2 cycles, 10 seconds each. Effective ablation was achieved with intralesional hyperechoic signal noted after the procedure. (**Figure 3**) Fine needle biopsy (FNB) was also performed before RFA using a 19G Procore needle. However, the biopsy sample contained multiple pieces of blood embedded with scanty pancreatic acinar parenchyma and strips of benign ductal epithelium rendering it insufficient for pathological diagnosis. After the procedure, his capillary blood glucose readings rose to 15 – 16 mmol/L the following day with complete resolution of hypoglycaemic episodes. Real-time CGM (Dexcom, CLARITY) was repeated in the post-operative period which confirmed the absence of hypoglycaemia with persistent fasting and postprandial hyperglycaemia. The average glucose level was 19 mmol/L. The patient was initially started on twice daily premixed human insulin in addition to metformin for glycaemic control. His insulin regimen was gradually intensified to basal bolus therapy with insulin analogues for optimization. A reassessment contrast CT abdomen performed after 18 months showed complete resolution of the previously noted pancreatic head lesion. (**Figure 4**) Together with the fact that the patient remained insulin-requiring without significant hypoglycaemic attacks, complete cure of the insulinoma is achieved.

## Discussion

The co-existence of insulinoma and T2D is uncommon but has been reported in the literature. Notably, most of these cases had insulinoma presenting after the diagnosis of T2D, with recurrent hypoglycaemia despite dose reductions in insulin therapy and anti-diabetic agents. The time from presentation to the diagnosis of insulinoma ranged from 6 to 36 months (3, 7–10). There was also one report of a patient who was found to have a 5.5cm pancreatic head tumour within a month after the diagnosis of T2D. That patient did not have any clinical or biochemical evidence of spontaneous hypoglycaemia all along, although histological examination confirmed insulinoma (11). The diagnosis of insulinoma with coexisting diabetes is challenging. Patients with T2D often have insulin resistance, which may dampen the severity of hypoglycaemic symptoms and result in delayed presentation. Moreover, in patients with diabetes, even if hypoglycaemic symptoms are present, they are often attributed to iatrogenic causes such as the use of insulin therapy or insulin secretagogues. As a result, this delay in diagnosis may lead to a higher likelihood of progression of the underlying insulinoma to more advanced disease, larger tumour size and a higher rate of malignancy which has been reported as high as 25% compared to the usual quoted rate of 10% (5).

Early resection of insulinoma is crucial in the management of T2D in these patients. Since insulin secretion by the tumour is autonomous, it is often difficult to start any anti-diabetic medications in these patients due to the presence of recurrent hypoglycaemia. On the other hand, although diazoxide is frequently used in the usual patients with insulinoma for alleviation of hypoglycaemic symptoms while awaiting tumour resection, it may worsen glycaemic control in these rare patients with co-existing T2D, since diazoxide inhibits insulin release through targeting potassium channels in the pancreatic beta islet cells.

RFA is a widely used technique for treating dysplastic or neoplastic tissue through the induction of local thermal coagulative necrosis (6). However, RFA of pancreatic lesions was less commonly performed, likely related to concerns about pancreatic tissue being more sensitive to thermal injury. Moreover, the proximity of pancreas to surrounding vasculature and biliary system also carries risks of structural injury. In this regard, endoscopic and intraoperative approaches were described to be relatively safer than the percutaneous approach. However, intraoperative RFA was still invasive, and transabdominal ultrasound-guided percutaneous RFA is often limited by poor visualization of the retroperitoneal pancreas. Therefore, EUS allows both localization of the pancreatic lesion and provision of a real-time guidance during the RFA procedure. Studies in animals and human illustrated the role of EUS-guided RFA as a feasible and safe procedure for pancreatic lesions (6). An early study that compared intraoperative, percutaneous and EUS-guided RFA on a small number of patients with pancreatic NET (PNET) demonstrated that EUS-guided RFA could achieve rapid and complete normalization of serum hormone levels without recurrence observed over a median follow-up of 34 months (12). In another case series that evaluated the application of EUS-guided RFA among 3 patients with insulinoma who refused or were contraindicated for surgery, symptomatic relief was achieved within 24 hours after the procedure. In our patient, the complete resolution of the pancreatic head insulinoma on reassessment CT scan 18 months after RFA and the continued clinical remission suggest a lasting effect of RFA. Moreover, the procedure was overall well tolerated except for mild abdominal pain (13), which is a commonly reported adverse effect of EUS-guided RFA in addition to the transient, asymptomatic rise in serum amylase and lipase levels. A recent review has proposed several important factors to consider when evaluating PNET patients for EUS-RFA, which include the functionality, number, size, grade and stage of the tumours (14). In properly selected patients, EUS-guided RFA provides a less invasive alternative therapeutic strategy and spares these patients from pancreatic surgery, which has been associated with both morbidity and mortality, including postoperative hemorrhage (1-7%), pancreatic fistula (14-58%), delayed gastric emptying (5-18%) and in-hospital mortality (3-6%) (14, 15).

Despite the aforementioned benefits of EUS-RFA, this treatment modality is not without limitations. In patients with functioning pancreatic PNET (F-PNET), the resolution of clinical symptoms and biochemical abnormalities is often diagnostic of cure. Routine histological diagnosis is hence not commonly required for patients with insulinoma especially when pre-operative DOTATATE scan also does not reveal any metastasis, as in our patient (14) (16). However, in patients with non-functioning PNET (NF-PNET), accurate tumour diagnosis and grading on biopsy specimens are crucial for proper treatment planning. Accordingly, the lack of histological diagnosis in EUS-RFA, as opposed to conventional surgery, is a potentially important limitation in these patients, although this could be resolved with pre-ablation EUS-guided fine needle aspiration (FNA) or FNB. In a recent study, satisfactory correlation in tumour grading and Ki-67 index has been found between cytology samples obtained from EUS-guided FNA and histological samples from pancreatic resection (17). A meta-analysis further demonstrated that EUS-guided FNB provided even superior sample adequacy and diagnostic accuracy over FNA, with similar complications rate and technical success (18). Nonetheless, inadequate sampling may still occur with fine needle-based technique as in our patient. Another limitation is the lack of standardized imaging protocol to assess the completeness of ablation. There has been concerns that residual or recurrent tumours may be difficult to be distinguished from post-procedural inflammation and necrosis on reassessment imaging.

Our case also highlighted the usefulness of applying CGM in these patients. Indeed, the increased availability and affordability of different CGM systems in recent years have benefited patients with diabetes who have glycaemic fluctuations, as well as clinicians especially during the diagnostic workup of these rare conditions. Moreover, CGM systems with alert function during severe hyperglycaemia or hypoglycaemia could also help trigger timely corrective measures and improve patient safety while awaiting definitive treatment for the insulinoma in the background of concomitant diabetes.

## Conclusion

This case illustrates the challenges in the diagnosis of an insulinoma presenting simultaneously with T2D. Clinical vigilance is essential in identifying these two apparently contradictory conditions. EUS-guided RFA provides a real-time, image-guided, safe and minimally invasive option of insulinoma ablation. However, while this treatment modality can be considered in patients with insulinoma especially those who refuse or are contraindicated for pancreatic surgery, clinicians should also be aware of its limitations especially when histological confirmation is necessary for further clinical management.

## Data Availability Statement

The raw data supporting the conclusions of this article will be made available from the corresponding author on reasonable request.

## Ethics Statement

Written informed consent was obtained from the individual(s) for the publication of any potentially identifiable images or data included in this article.

## Author Contributions

JC and CW researched the data and wrote the manuscript. DL, MF, AL, EL, Y-CW, W-SC, KL, KT and C-HL critically reviewed and edited the manuscript. C-HL initiated and conceptualized this case report and is the guarantor of this work. All authors contributed to the article and approved the final manuscript. All authors contributed to the article and approved the submitted version.

## Conflict of Interest

The authors declare that the research was conducted in the absence of any commercial or financial relationships that could be construed as a potential conflict of interest.

## Publisher’s Note

All claims expressed in this article are solely those of the authors and do not necessarily represent those of their affiliated organizations, or those of the publisher, the editors and the reviewers. Any product that may be evaluated in this article, or claim that may be made by its manufacturer, is not guaranteed or endorsed by the publisher.
